# Peptidylarginine Deiminases—Roles in Cancer and Neurodegeneration and Possible Avenues for Therapeutic Intervention via Modulation of Exosome and Microvesicle (EMV) Release?

**DOI:** 10.3390/ijms18061196

**Published:** 2017-06-05

**Authors:** Sigrun Lange, Mark Gallagher, Sharad Kholia, Uchini S. Kosgodage, Mariya Hristova, John Hardy, Jameel M. Inal

**Affiliations:** 1 Department of Biomedical Sciences, University of Westminster, 115, New Cavendish Street, London W1W 6UW, UK; 2 School of Pharmacy, University College London, 29-39 Brunswick Square, London WC1N 1AX, UK; 3 Cellular and Molecular Immunology Research Centre, School of Human Sciences, London Metropolitan University, 166-220 Holloway Road, London N7 8DB, UK; m.gallagher@londonmet.ac.uk (M.G.); uck0002@my.londonmet.ac.uk (U.S.K.); j.inal@londonmet.ac.uk (J.M.I.); 4 Molecular Biotechnology Center, Department of Medical Sciences, University of Turin, Corso Dogliotti 14, 10126 Turin, Italy; sharad.kholia@gmail.com; 5 Institute for Women’s Health, University College London, 74 Huntley Street, London WC1N 6HX, UK; m.hristova@ucl.ac.uk; 6 Reta Lila Weston Research Laboratories, Department of Molecular Neuroscience, UCL Institute of Neurology, London WC1N 3BG, UK; j.hardy@ucl.ac.uk

**Keywords:** extracellular vesicles (EVs), microvesicles (MVs), exosomes, peptidylarginine deiminases (PADs), deimination, Chlor-amidine (Cl-Am), cancer, neurodegeneration, cytoskeleton, induced pluripotent stem cells (iPSCs), histone H3, epigenetics

## Abstract

Exosomes and microvesicles (EMVs) are lipid bilayer-enclosed structures released from cells and participate in cell-to-cell communication via transport of biological molecules. EMVs play important roles in various pathologies, including cancer and neurodegeneration. The regulation of EMV biogenesis is thus of great importance and novel ways for manipulating their release from cells have recently been highlighted. One of the pathways involved in EMV shedding is driven by peptidylarginine deiminase (PAD) mediated post-translational protein deimination, which is calcium-dependent and affects cytoskeletal rearrangement amongst other things. Increased PAD expression is observed in various cancers and neurodegeneration and may contribute to increased EMV shedding and disease progression. Here, we review the roles of PADs and EMVs in cancer and neurodegeneration.

## 1. Introduction

Exosomes and microvesicles (EMVs) play physiological roles as mediators of intercellular communication, transferring molecules characteristic of their parental cells such as receptors, enzymes, cytokines, growth factors, and genetic material—including miRNAs—to recipient cells thus affecting diverse processes such as differentiation, migration, and angiogenesis [1,2,3,4]. As EMVs are present in body fluids including blood, urine, and cerebrospinal fluid, they may serve as reliable biomarkers of pathophysiological processes [5,6,7,8,9].

Microvesicles (MVs), are 100–1000 nm sized phospholipid-rich vesicles that are released from the cell membrane of diverse cell types as part of normal cell physiology [10,11] and upon stimulation, with for example growth factors or cytokines, and/or in early apoptosis or [5,7]. MV release depends on calcium ion influx, which occurs either through pores created by sublytic complement or stimulation of calcium channels, such as P2X_7_, or calcium released by the endoplasmic reticulum through various calcium channels on activated cells [3,12]. MVs can also be released during pseudoapoptotic events [13]. The increase in cytosolic calcium results in cytoskeletal reorganization which is facilitated by the activation of various enzymes, including calpain, gelsolin, and scramblase; protein kinase ROCK-1 (Rho associated kinase 1) and the simultaneous inhibition of translocase and phosphatases [14]. Subsequent loss of membrane asymmetry and membrane blebbing leads to MV formation and release [6,15].

Exosomes are smaller than MVs, 30–100 nm in size, are generated intracellularly, and released into the lumen of an endosome that becomes a multivesicular body which then is exocytosed, releasing its cargo of exosomes at the plasma membrane [12,16,17]. Cellular components crucial for exosome formation include components of ESCRT, which are involved in the formation of multivesicular bodies (MVBs) and intraluminal vesicles [18,19]; syntetin and syndecan [20]; sphingolipid ceramide and tetraspanins [21]. Exosome secretion can also be modulated by microenvironmental pH [22]. During the final step of exosome release, the multivesicular bodies fuse with the plasma membrane, mediated by membrane-bridging SNARE complex machinery [23], which has been reported to participate in the fusion between MVBs with the plasma membrane and exosomal release into the extracellular medium [24]. In addition to EMVs, larger vesicles (>1 µm) are released from cells as apoptotic bodies [25].

As EMVs have been shown to actively contribute to the progression of numerous pathologies —including cancers [10,26,27,28] and autoimmune [29,30] and neurodegenerative [8,31,32,33] diseases—they pose as therapeutic targets in treatment of disease. Unravelling mechanistic pathways involved in EMV biogenesis may thus provide avenues for selective interception of EMV release [5,7]. Recent discoveries have elucidated roles for peptidylarginine deiminases (PADs) and their pharmacological inhibition in EMV shedding [26,34]. The PADs are a family of five tissue specific calcium activated enzymes that cause irreversible changes of protein-bound arginines into citrullines [35,36], resulting in protein misfolding and functional changes in target proteins [36,37,38]. While PADs play physiological roles [39], their dysregulation is detected in various pathologies [40,41,42,43,44]. Pharmacological PAD-inhibition has shown promising results in cancer models both in vitro [45,46] and in vivo [43,47], as well as in animal models of various autoimmune diseases [48,49,50,51,52], neuronal injury [53], hypoxia [54], and atherosclerosis [55].

## 2. Exosomes and Microvesicles EMVs in Cancer

Cumulative evidence implicates EMVs in the pathogenesis of cancer, either directly or indirectly. Elevated EMV levels in the blood from cancer patients has been demonstrated by various investigators and been shown to aid tumour spread and survival [56,57,58]. EMV shedding from cancer cells can contribute to their resistance to chemotherapeutic agents and has been shown to increase active drug efflux. In addition, chemotherapeutic drugs have been shown to stimulate cells to release EMVs, which have been shown to carry the drugs within them [22,59,60,61,62,63,64,65,66]. It has been shown that inhibition of EMV release can effectively increase drug retention within cancer cells and render them more susceptible to anticancer drug treatment [22,27,60,61,62] as well as reducing the dose of docetaxel required to limit tumor growth in vivo [59].

## 3. Peptidylarginine Deiminases PADs in Cancer

PAD dysregulation is elevated in numerous malignant tumours and associated with cancer progression. Overexpression of PAD2 and PAD4 isozymes has been reported in patients’ blood and tissues [67,68,69,70,71].

PAD4 is the only isozyme that contains a classic nuclear localisation signal [72,73] and acts as a transcriptional co-regulator for various factors including p53, p300, p21, and ELK1 and via deimination of the N-terminal tails of various histone proteins [74,75,76]. PAD4 plays a role in apoptosis as it regulates p53 gene activity during DNA damage by acting as a co-mediator of gene transcription and epigenetic cross talk with histone deacetylase 2 (HDAC2) [77]. PAD4 is also co-localised with cytokeratin (CK), an established tumour marker which occurs in various isoforms, some of which are deiminated. The deiminated CK isoforms become resistant to caspase-mediated cleavage, contributing to the disruption of apoptosis in cancer tumours [68]. PAD4 also acts as a cofactor in epidermal growth factor-mediated target gene activity, activating the expression of proto-oncogene c-fos [76], interacting with p53 and influencing the expression of its target genes [74,75,78,79]. PAD4 is also linked with oestrogen receptor target gene activity via histone tail deimination [80]. In gastric carcinoma, PAD4 upregulates C-X-C chemokine receptor 2 (CXCR2), keratin 14 (KRT14) and tumour necrosis factor-α (TNF-α) expression levels [81].

Both PAD2 and PAD3 have also been localized and detected in the nucleus in spite of lacking a classic nuclear translocation site such as is found in PAD4 [54,70,82]. In cancer cells, PAD2, which is the most widely expressed isozyme in the body [35], has been shown to deiminate histone H3 and play a role in gene regulation [43,70,83,84]. Recent studies are increasingly identifying multifactoral roles for PAD2 and PAD4 in cancer pathologies, depending on tumour type [71,85,86,87,88]. In gastric cancer, the *PADI2* gene was found to advance abnormal cell behaviour by increasing expression levels of CXCR2, a cell proliferation and invasion gene; while *PADI2* has deleterious effects on tumour growth and metastasis in liver tumour cells via regulation of the tumour growth gene erythropoietin (EPO) [71]. Colon cancer has, on the other hand, been associated with downregulation of *PADI2* [86,87], while *PADI2* affects differentiation of normal colon and can suppress proliferation of colonic epithelial cells through protein deimination [86,87], accompanied by arrest of cell cycle progression in G1 phase [86]. In colon cancer cells (HCT116), PAD-inhibitor Cl-amidine induces the upregulation of several tumor suppressor microRNAs, which are otherwise downregulated in cancers [89]. In breast cancer (MCF-7 cells), inhibiting *PADI2* expression significantly decreased cell migration ability but did not affect cell proliferation and apoptosis [85]. PAD4 has also been shown to negatively regulate tumor invasiveness in breast cancer models both in vitro and in vivo via citrullination of glycogen synthase kinase-3β (GSK3β) [88]. Overall, these findings emphasize the need for further testing of PAD isozyme selective inhibitors for intervention in cancer, alone or in combination, with regard to tumour type.

## 4. The Interplay of PADs and EMVs in Cancer

The presence of PADs has been confirmed in EMVs released from various cancers cells [90]. Based on a search in the Vesiclepedia dataset (http://www.microvesicles.org/), using gene symbol identifiers, PADs have been reported in EMVs from melanoma, breast, colon, kidney, lung, melanoma, ovarian, and prostate cancer cell lines [90], as well as colorectal cancer cells [91]. It may be postulated that the increased EMV release observed in cancers is partly driven by elevated PAD expression in cancers and that PAD enzymes—which are amongst the cargo packaged in EMVs—are carried into plasma where they can deiminate target proteins [92]; and aid in the spread of cancer indirectly.

In metastatic prostate PC3 cancer cells, both PAD2 and PAD4 isozymes were found to be elevated and to undergo increased nuclear translocation in correlation with increased EMV release [26].

Both PAD2 and PAD4 have been shown to translocate to the nucleus in response to TNFα upregulation [93,94,95]. As part of the inflammatory response, it may be postulated that increased EMV release also causes upregulation of TNFα which may lead to a feed-back loop of PAD translocation and EMV shedding in an ongoing inflammatory environment.

Which of the PAD isozymes is the main player in EMV release and the critical respective target proteins for successful MV and/or exosome shedding has to be further investigated. The different PADs may well be either selectively or collectively involved with different, albeit equally important, roles. In addition, the specific effect of PAD isozymes involved in EMV biogenesis will need to be taken into consideration dependent on tumour type. The selectivity of potential EMV inhibitors and combinatory application with chemotherapeutic agents is thus of great interest. Most potential EMV inhibitors tested so far have displayed a preferential tendency for inhibition of either MVs or exosomes [22,34,59,61,96,97,98] and thus the effect of PAD inhibitor Cl-amidine observed on both vesicle types indicates their potential usefulness. A combination of selective EMV inhibitors may indeed encourage re-testing of chemotherapeutic drugs currently not in favour due to severe side effects and poor effectiveness, as for example 5-FU treatment of prostate cancer [99].

## 5. Deiminated Target Proteins and PAD-Interacting Proteins Identified in EMV Biogenesis

Depending on target protein preference of PAD2 and PAD4, EMV release may occur via cytoskeletal and/or epigenetic pathways as the different PAD isozymes have indeed demonstrated distinct substrate preferences, with PAD4 showing more restrictive substrate specificity compared to PAD2 [100,101,102,103]. While PAD4 prefers sequences with highly disordered conformation, PAD2 has a broader sequence specificity, which might partly be reflected by the broader tissue expression of PAD2 [104]. PAD2 deiminates β- and γ-actins [100] and has been shown to affect histone H3 deimination [84], while PAD4 has been shown to deiminate histone H3 and H4 [104,105] and to regulate histone arginine methylation levels [80].

**Figure 1:**
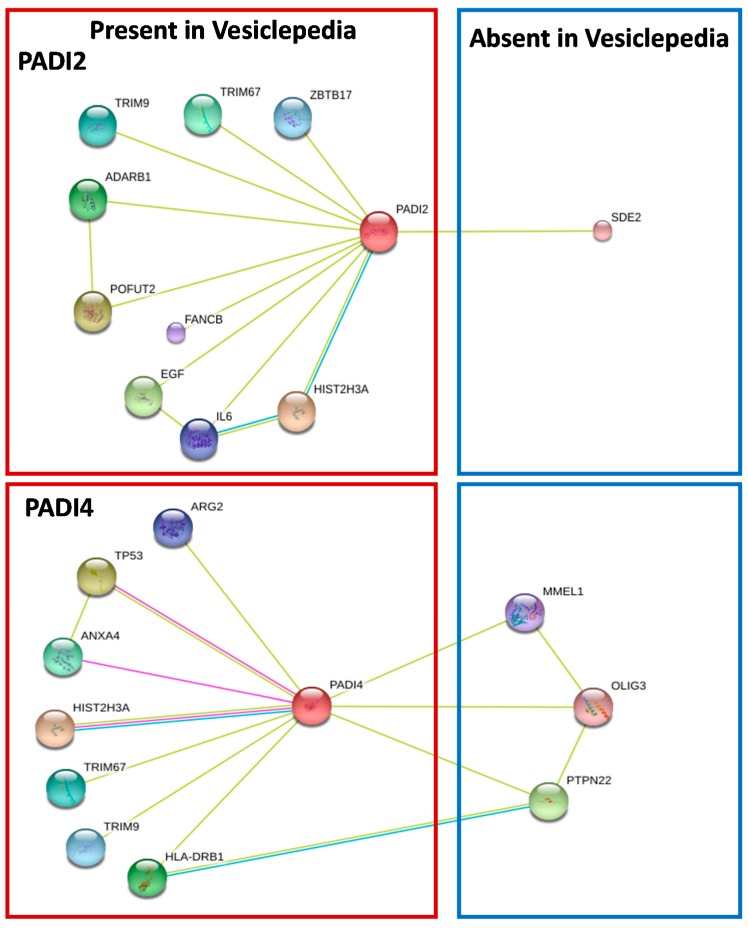
STRING analysis (https://string-db.org/) showing putative binding partners (STRING combined score >0.4) of PAD2 and PAD4, identified and found to be present in exosomes and microvesicles EMVs based on a search by gene symbol in the Vesiclepedia protein data set. Lines between nodes represent the following: Green line = text mining; Blue line = from curated database; Pink line = experimentally determined.

Targets of PAD-activation observed in EMV release include cytoskeletal actin which contributes to the reorganisation of the cytoskeleton necessary for successful vesicle release [15]. The presence of deiminated β-actin increased in cells that were stimulated for EMV release was markedly diminished after pre-treatment with PAD-inhibitor [26]. β-Actin, one of six different human actin isoforms, is a cytoskeletal protein involved in cell structure and integrity, cell migration, and movement [106]. This provides evidence for the importance of PAD-mediated deimination of target proteins that are involved in cytoskeletal rearrangement—such as β-actin, actin α1, and glyceraldehyde-3-phosphate dehydrogenase (GAPDH)—as an essential step for successful EMV biogenesis as the process of multivesicular body recruitment to the plasma membrane to release exosomal cargo likely involves actin and microtubular elements of the cytoskeleton [107]. During vesicle formation, both β- and F-actin stress fibres play important roles in the redistribution of the actin-cytoskeleton through the activation of Rho/Rho-associated kinase (ROCK) pathways during apoptosis and thrombin stimulation [14]. Deiminated β- and γ-actins have indeed also previously been detected in sera and synovial fluid from RA patients [108] and been identified as a substrate for PAD2 in ionomycin-activated neutrophils [100]. Other deiminated protein targets identified in association with EMV release included GAPDH, which is reported to be exosome associated ([109] http://www.exocarta.org). It is a multifunctional enzyme involved in glycolysis, nuclear functions such as transcription and DNA replication, as well as apoptosis [110]. GAPDH has also been shown to contribute to the regulation of intracellular Ca^2+^ levels via binding to integral membrane proteins, such as the inositol-1,4,5-triphosphate receptor (IP3R) and sarcoplasmic reticulum Ca^2+^ (SERCA) pump [111,112]. Cytosolic GAPDH also catalyzes microtubule formation and polymerization by binding the cytoskeletal protein tubulin [113] and is associated with endoplasmic reticulum (ER) to Golgi vesicular transport [114]. Based on a STRING analysis (https://string-db.org/), putative binding partners of *PADI2* and *PADI4* were identified and found to be present in EMVs based on a search by gene symbol in the Vesiclepedia protein data set (Figure 1). These included histone H3, known to be deiminated [84,104,105,115]; p53, which is known to be regulated by PAD4 [74,116]; interleukin 6 (IL6), one of the major cytokines in the tumour microenvironment [117]; epidermal growth factor (EGF) which is a crucial mitogenic factor including in prostate cancer [118]; Tripartite Motif Containing (TRIM) 9 and TRIM 67 which are associated to microtubule binding [119], lung cancer [120], and neuronal differentiation [121]; Arginase 2 (ARG2), which has roles in suppressing macrophage cytotoxicity and myeloid-derived suppressor cell function [122] and is elevated in breast cancer [123]; Zinc-finger and BTB domain-containing protein 17 (ZBTB17/Miz1) which modulates Myc, a multifunctional nuclear phospoprotein in cell cycle progression, apoptosis, and cellular transformation and which is enhanced in tumours [124]; Adenosine Deaminase, RNA Specific B1 (ADARB1), which is overexpressed in various cancer cell types and transformed stem cells [125]; Annexin A4 (ANXA4), the upregulation of which promotes the progression of tumour and chemoresistance of various cancers [126]; Major histocompatibility complex, class II (HLA-DRB1), which besides known functions in autoimmunity, including the generation of anti-citrullination antibodies [127], is also associated to carcinoma [128].

## 6. PADs in Central Nervous System (CNS) Damage and Neuroprotective Effects of PAD Inhibitors

In two animal models of acute CNS damage, pharmacological pan-PAD inhibition has been shown to be neuroprotective in vivo following administration straight after insult and for up to two hours post-injury, indicating a clinically relevant time window for intervention [53,54,129]. Firstly, in a spinal cord injury model, significant reduction was observed in infarct size, accompanied by reduced neuronal cell death and histone H3 deimination, compared to non-treated control injuries [53]. Secondly, two murine models of neonatal hypoxic ischaemic encephalopathy (HIE), showed similar neuroprotective effects as estimated by volume infarct analysis, reduced cell death, and histone H3 deimination, and in addition a significant impact on neuroinflammatory responses as reflected in reduced microglial activation in all affected brain regions [54]. The fact that these neuroprotective effects of PAD-inhibitors are translatable between CNS injury and animal models, is indeed promising for effective application also in other cases of neuronal damage. Interestingly, while increased protein deimination has been also detected in the pathology of traumatic brain injury [130], EMV release has been associated with cerebral hypoxia induced by acute ischaemic stroke [131,132] and mesenchymal stromal cell-derived EMVs have recently been shown to protect the foetal brain following hypoxia-ischaemia in an experimental ovine model [133], and to be neuroprotective in stroke [134,135] and traumatic brain injury [136] rat models. The significance of EMV release in relation to pharmacological PAD manipulation requires further investigation in acute CNS damage.

## 7. EMVs in Neurodegenerative Diseases

EMVs are increasingly being associated with neurodegenerative disease progression and pathologies [137,138,139,140,141,142,143]. In the CNS, EMVs have been shown to be produced by several cell types including neurones, microglia, oligodendrocytes, astrocytes, and embryonic neural stem cells [8,144,145,146] and to play important roles in the development and function of the nervous system [147]. Roles for EMVs in neurodegenerative disease progression include intercellular communication and neuroinflammation due to transport of parent-cell specific cargo that can be translated in recipient cells and also affect gene regulation [148,149,150]. In Amyotrophic Lateral Sclerosis (ALS), exosomes have for example been shown to export misfolded mutant superoxide dismutase 1 (SOD1) [151,152]; in relation to ALS and Frontotemporal dementia (FTD) to export TAR DNA-binding protein 43 (TDP-43) [153,154]; and there is increasing evidence emerging for critical roles for miRNA transport in the pathogenesis of FTD-ALS [155,156]. In tauopathies, EMVs have been shown to export phosphorylated tau [157,158]; in Parkinson’s disease (PD), exosomes were shown to export α-synuclein and leucine-rich repeat kinase 2 (LRRK2) [159,160,161]; and in Alzheimer’s disease (AD), they export amyloid β (Aβ) [162,163]. All of these proteins form aggregates involved in the disease pathologies [164]. As EMVs have the capability to travel further via the blood or cerebrospinal fluid, misfolded proteins may spread via this pathway in a prion-like manner [165,166,167,168,169,170]. In addition, functional effects of such a protein transport have been indicated for Aβ, which progressively accumulates in EMVs with age, while the β-site cleavage of amyloid precursor protein (APP) has been reported to occur inside EMVs [171]. Also, the phosphorylation of tau differs in exosomes compared to total cell lysates, indicating functional consequences for its seeding capability [157]. In AD, neuroinflammation has been linked to circulating TNFα [172,173,174], which causes nuclear translocation of PADs [94,95], and to neutrophil extracellular trap formation [175], which is PAD4-dependent [38,94] and causes externalization of deiminated histones [176] and release of active PAD enzymes [177]. In addition, in PD, α-synuclein induces TNF-α containing exosomes from microglia [161] while TNF-α has been shown to promote EMV shedding from endothelial cells [162]. In light of this increasing evidence for crucial roles of EMVs in neuroinflammation, and the transfer and spreading of neurodegenerative protein aggregates alongside other cargo, the mechanisms of EMV biogenesis and routes of modulation are pivotal. It has also to be considered that the primary changes in most neurodegenerative diseases occur in specific brain locations followed by propagation into well-defined brain regions. The levels of secretion and cargo composition may thus not be homogenous among brain regions [142].

## 8. PADs and Protein Deimination in Neurodegenerative Diseases

The evidence for critical roles of PADs in various neurodegenerative diseases is mounting [178,179,180,181,182,183]. A human RNA-Seq transcriptome and splicing database of glia, neurones, and vascular cells of the cerebral cortex shows highest levels of *PADI2* in mature astrocytes, oligodendrocytes, and microglia [184]. In many cases where protein deimination has been associated with neurodegenerative diseases, including multiple sclerosis (MS) [185,186,187,188], AD, and PD, studies have mainly focused on histological analysis of post mortem human samples. AD post mortem human brain samples display increased protein deimination [179,180,181,189,190,191] and deiminated proteins are present in amyloid-containing areas in amyloid-precursor-protein/presenilin1 (APP + PSEN1) transgenic AD mouse models [44,192].

Although some deiminated target proteins have been described, most remain to be identified. Using proteomic analysis of deiminated proteins in the injured CNS, several proteins with neurodegenerative implications were identified, including with roles in neuroinflammation and perivascular drainage of Aβ [53,54,193]. In AD patients, β-amyloid has been shown to be deiminated [44,181]. In hippocampal lysates from AD patients, glial fibrillary acidic protein (GFAP), an astrocyte-specific marker protein, and vimentin were identified as deiminated proteins and the deimination of GFAP was shown to be PAD2 specific [194]. In vitro studies demonstrated that amyloid peptides bind to PAD2, resulting in catalytic fibrillogenesis and formation of insoluble fibril aggregates [42]. In PD brain samples, increased levels of total protein deimination and deimination-positive extracellular plaques were observed [178]. Mutated misfolded α-synuclein protein has been related to increased protein deimination, amyotrophic lateral sclerosis (ALS) spinal cords show increase in deiminated proteins [44], and Creutzfeldt Jacob Disease (CJD) brain samples indicate roles for deiminated enolase [195]. In AD brains, pentatricopeptide repeat-containing protein 2 (PTCD2), a mitochondrial RNA maturation and respiratory chain function protein [196], is present in a deiminated form and is an antigen target of an AD diagnostic autoantibody. There are thus indications that disease-associated autoantibodies are generated due to the production and release of deiminated proteins and deiminated protein fragments, which may be released from damaged cells in regions of pathology [197,198]. In AD, both PAD2 and PAD4 were shown to be expressed in cerebral cortex and hippocampus, the brain regions most vulnerable to AD pathology, with PAD2 localized in activated astrocytes and PAD4 selectively expressed in neurones [197]. Evidence for increased PAD expression with progression of neurodegenerative disease has also been obtained by analysis of whole genome microarrays from mouse models carrying TAU and APP+PSEN1 mutations. Significant increase of *PADI2* transcription was found in cortex and hippocampus in both mutants with disease progression compared to age matched controls [193]. PAD4 expression has been shown to co-localize with amyloid-β-42 in pyramidal neurones in cerebral cortex and in hippocampal large hilar neurones of the hippocampus, which were also surrounded by activated astrocytes and microglia. These neurones contained cytoplasmic accumulations of deiminated proteins [197]. Using iPSC neuronal models derived from fibroblasts from patients [199] carrying FTD/ALS associated valosin-protein containing mutations *VCP*R155C and *VCP*R191Q, both PAD2 and PAD4 expression, accompanied by significantly increased pan-protein deimination, has been observed compared to control (non-mutation carrying) neurones, with significant increases in histone H3 deimination in *VCP*R155C carrying neurones [193]. Similar changes were also observed for α-synuclein triplication [200] compared to control neurones [193]. The release of deiminated proteins from necrotic neurones has been thought to cause an increased exposure of deiminated neuronal proteins to the immune system. In addition, the continual return of cerebrospinal fluid to circulation via the arachnoid villi, containing modified deiminated proteins and protein fragments, has been suggested to be a key step in the ongoing pathology due to generation of autoantibodies [197]. PADs are thus expressed in neurones residing in brain regions that are engaged in neurodegenerative pathological changes and inflammatory changes such as reactive astrogliosis and microglial migration and invasion. This brain-region specific increase observed in PAD expression may affect local exosome or microvesicle release specifically, contributing to spread of pathology in these regions.

**Figure 2:**
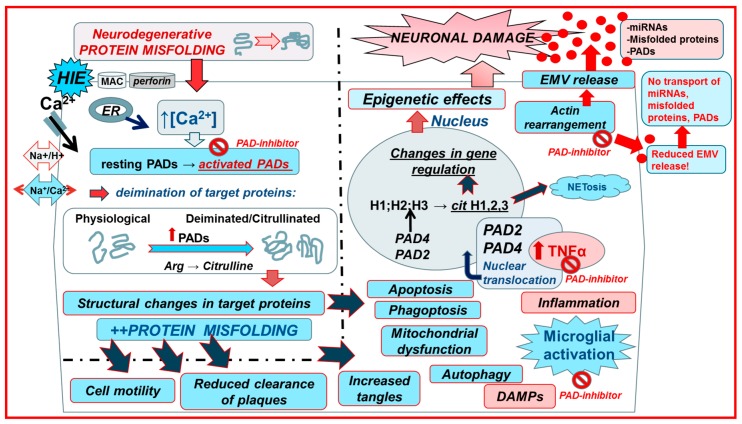
Mechanisms of peptidylarginine deiminases PADs) in central nervous system (CNS injury and neurodegenerative pathologies and the proposed effect of PAD-inhibitors. Upon CNS injury (hypoxic ischaemic encephalopathy, HIE), Ca^2+^ entry is facilitated via the reversal of the Na^+^/Ca^2+^ exchanger due to over activation of the Na^+^/H^+^ exchanger (NHE). Ca^2+^ entry can also be facilitated due to membranolytic pathways including the complement membrane attack complex (MAC) and perforin. Increased cytosolic Ca^2+^ triggers the neurotoxic cascade, which includes activation of the Ca^2+^ dependent PAD enzymes. Neurodegenerative disease mutations cause protein aggregation and impaired calcium buffering, which activates the downstream PAD-cascade. Both in CNS acute injury and neurodegeneration, PAD activation causes protein deimination and further protein misfolding, affecting cell motility, autophagy, phagoptosis, and mictochondrial function, leading to neurotoxic events. Deiminated neo-epitopes and leakage of deiminated proteins from dying cells contribute to neuroinflammation that in turn may upregulate TNFα, which causes nuclear translocation of PADs, leading to histone deimination and also formation of neutrophil extracellular traps (NETosis). PAD-mediated cytoskeletal protein deimination and nuclear PAD translocation, which can affect histone deimination, contribute to EMV release, resulting in export of misfolded proteins, DNA, RNA, miRNAs, enzymes, and other EMV cargo that can contribute to pathologies. PAD-inhibitior Cl-Amidine targets PAD activation and reduces deimination of target proteins and neuroinflammatory responses. Cl-Amidine also significantly reduces EMV shedding, resulting in decreased transport of noxious EMV cargo (red arrows emphasise the main events associated to PAD activation and PAD inhibition that affect EMV release. Blue arrows indicate additional downstream changes due to PAD-mediated protein misfolding; based on [26,129]).

Figure 2 summarises the proposed interplay of PADs and EMVs in neurodegenerative disease pathologies.

## 9. Conclusions

Recent studies have emphasized roles for both EMVs and PAD enzymes in cancers and neurodegeneration. Critical roles for PADs and their pharmacological inhibition have been established in cancers and neuroinflammation. PAD-mediated mechanisms have been shown as a novel mediator in the biogenesis of EMVs, which may contribute in part to increasing EMV shedding from cancer cells and act as a protective mechanism to expel chemotherapeutic drugs. In the context of neurodegeneration, EMVs are increasingly implicated in the spread of pathologies via transfer of miRNAs and misfolded proteins. While Cl-amidine [201] remains the most used experimental pan-PAD inhibitor to date, the therapeutic potential and generation of second generation and selective isozyme-specific PAD inhibitors is receiving ever increasing attention [45,49,96,202,203,204,205,206,207]. The use of targeted isozyme-selective PAD inhibitors in synergy with other EMV modulators—aimed at either exosomes, MVs, or both populations in conjunction—present promising combinatory therapies for both cancers and neurodegenerative diseases.

## Abbreviations

AD	Alzheimer’s disease
ALS	Amyotrophic lateral sclerosis
APP	Amyloid precursor protein
CJD	Creutzfeldt Jacob Disease
CK	Cytokeratin
Cl-Am	Chlor-amidine
CNS	Central nervous system
EV	Extracellular vesicle
FTD	Frontotemporal dementia
GFAP	Glial fibrillary acidic protein
HDAC2	Histone deacetylase 2
HIE	Hypoxic ischaemic encephalopathy
iPSC	Induced pluripotent stem cell
LPS	Lipopolysaccharide
LRRK2	Leucine-rich repeat kinase 2
MAC	Membrane attack complex
MS	Multiple sclerosis
MV	Microvesicle
NET	Neutrophil extracellular trap
PAD	Peptidylarginine deiminase
PC3	Prostate cancer cell line
PD	Parkinson disease
PNT2	Control benign prostate cell line
PSEN1	Presenilin 1
RA	Rheumatoid arthritis
ROCK	Rho/Rho-associated kinase
SNARE	SNAP (Soluble NSF Attachment Protein) REceptor
SOD1	Superoxide dismutase 1
TDP-43	TAR DNA-binding protein 43
TNFα	Tumour necrosis factor α
VCP	Valosin containing protein
